# Supplementary material for: Identification of photoperiod-induced specific miRNAs in the adrenal glands of Sunite sheep (Ovis aries)
Source: Front Vet Sci. 2022 Jul 22;9:888207. doi: 10.3389/fvets.2022.888207 (PMC9354845; doi:10.3389/fvets.2022.888207)
Supplement: Supplementary file 1 [file Data_Sheet_1.ZIP › Supplementary file/miRDeep2 file.zip!/miRDeep2 file/Novel_Structure/SP42A2/Novel_399.pdf]

Provisional ID	:	NC_019459.2_5980
Score total	:	1
Score for star read(s)	:	-1.3
Score for read counts	:	0
Score for mfe	:	0.7
Score for randfold	:	1.6
Score for cons. seed	:	
Total read count	:	12
Mature read count	:	12
Loop read count	:	0
Star read count	:	0

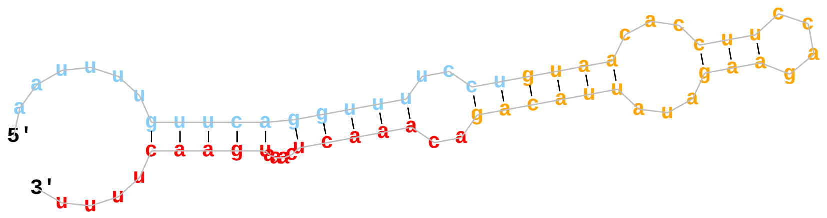

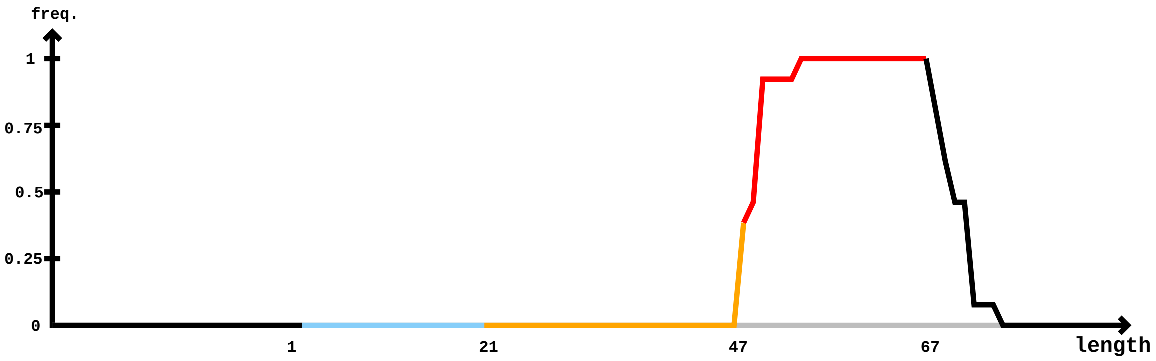


```
                                       Star                                            Mature
5'- gagagaauuauugaguuggccaaaaauuuuguucagguuuuccuguaacaccuuccagaagauauuacagacaaacucaaaugaacuuuuuggccagugcaauauaugcc   -3'    exp
    ........(((((..(((((((((((.((((((..(((((..((((((...(((....)))...))))))..)))))..)))))).)))))))))))..)))))......   reads    mm    sample
    ......................................................................aGaaacucaaaugaacuuuu....................   1        1     seq
    ......................................................................acaaacucaaaugaacuuuu....................   3        0     seq
    ......................................................................acaaacucaaaugaacuuuuug..................   1        0     seq
    .......................................................................caaacCcaaaugaacuuuuu...................   1        1     seq
    ........................................................................aaacucaGaugaacuuuuug..................   1        1     seq
    ........................................................................aaacCcaaaugaacuuuuuggc................   1        1     seq
    ........................................................................aaacAcaaaugaacuuuuuggc................   2        1     seq
    ........................................................................aaacucaaaugaacuuuuuggU................   2        1     seq
    ............................................................................ucaaaugaacuuuuuggccaU.............   1        1     seq
```
